# Selective intraarterial radionuclide therapy with Yttrium-90 (Y-90) microspheres for unresectable primary and metastatic liver tumors

**DOI:** 10.1186/1477-7819-9-86

**Published:** 2011-08-06

**Authors:** Ozlem N Kucuk, Cigdem Soydal, Seda Lacin, Elgin Ozkan, Sadik Bilgic

**Affiliations:** 1 Department of Nuclear Medicine, Faculty of Medicine, Ankara University, Ankara, Turkey; 2 Department of Radiology, Faculty of Medicine, Ankara University, Ankara, Turkey

**Keywords:** Selective intraarterial radionuclide therapy (SIRT), liver tumors, survival times

## Abstract

**Background:**

The aim of this study was to evaluate the success of selective intraarterial radionuclide therapy (SIRT) with Yttrium-90 (Y-90) microspheres in liver metastases of different tumors. We also interpreted the contribution of SIRT to survival times according to responder- non responder and hepatic- extra hepatic disease.

**Methods:**

The clinical and follow-up data of 124 patients who were referred to our department for SIRT between June 2006 and October 2010 were evaluated retrospectively. SIRT has been applied to 78 patients who were suitable for treatment. All the patients had primary liver tumor or unresectable liver metastasis of different malignancies. The treatment was repeated at least one more time in 5 patients to the same or other lobes. Metabolic treatment response evaluated by fluorine-18 fluorodeoxyglucose (F18-FDG) positron emission tomography/computed tomography (PET/CT) in the 6^th ^week after treatment. F18-FDG PET/CT was repeated in per six weeks periods. The response criterion had been described as at least 20% decrease of SUV value. Also in patients with neuroendocrine tumor serial Gallium-68 (Ga-68) PET/CT was used for evaluation of response. Patients were divided into 2 groups according to their treatment response.

**Results:**

68 patients received treatment for the right lobe, seven patients received treatment for the left lobe and 3 patients for both lobes. The mean treatment dose was estimated at 1.62 GBq. In the evaluation of treatment response; 43(55%) patients were responder (R) and 35 (45%) patients were non-responder (NR) in the sixth week F18-FDG PET/CT. Mean pretreatment SUVmax value of R group was 11.6 and NR group was 10.7. While only 11 (31%) out of 35 NR patients had H disease, 30 (69%) out of 43 R patients had H disease (p < 0.05). The mean overall survival time of R group was calculated as 25.63 ± 1.52 months and NR group's 20.45 ± 2.11 (p = 0.04). The mean overall survival time of H group was computed as 25.66 ± 1.52 months and EH group's 20.76 ± 1.97 (p = 0.09).

**Conclusions:**

SIRT is a useful treatment method which can contribute to the lengthening of survival times in patients with primary or metastatic unresectable liver malignancies. Also F18-FDG PET/CT is seen to be a successful imaging method in evaluating treatment response for predicting survival times in this patient group.

## Background

Primary or metastatic tumors of the liver generally have poor prognosis and are responsible for the shortening of overall survival times. Radioembolization with Yttrium-90 (Y-90) labeled microspheres (SIR spheres) (SIRT) is a palliative treatment method which could be applied to patients with unresectable liver tumors [1-3]. SIRT, firstly had been developed for the use of the treatment of unresectable hepatocellular carcinoma patients. Since then it has been used for the treatment of liver metastasis of different cancers [4-7]. Radiopharmaceutical includes resin bases microspheres which are labeled as Y-90. The diameter of spheres is approximately 29-35 μm. Although the portal venous system supplies the majority of the blood flow of normal liver tissue, liver metastases obtain almost all their blood flow by the hepatic artery. This situation is the principle of SIRT. Y-90 labeled microspheres which are applied to the hepatic artery cause micro embolization in the hepatic arterioles. In addition, Y-90 has beta particles, 64 hours' half-life and a 2.4 mm tissue penetration. In this way, in addition to mechanical obstruction, 30-60 Gray radiation doses are delivered to tumor tissue associated with applied Y-90 doses [8]. As a result, the surrounding liver tissue is protected.

The aim of this study was to evaluate the success of SIRT with Y-90 microspheres in liver metastases of different tumors. We also interpreted the contribution of SIRT to survival times according to responder- non responder and hepatic- extra hepatic disease.

## Patients and method

### Patients

The clinical and follow-up data of 124 patients who were referred to our department for SIRT between June 2006 and October 2010 were evaluated retrospectively. SIRT has been applied to 78 patients who were suitable for treatment. Of the remaining 46 out of 124 patients, the treatment could not have been performed because of the main contraindications of SIRT such as bilirubin levels>2 mg/dl or 5 fold elevation of AST and ALT levels or albumin levels< 3 mg/dl or bulky tumor>70% of liver tissue. All the patients had unresectable liver metastasis of different malignancies (35/78 colorectal, 25/78 hepatocellular, 7/78 gastric, 4/78 breast, 1/78 malign melanoma, 1/78 pancreas, 1/78 renal cell, 1/78 esophagus and 3/78 neuroendocrine tumor patients). All the patients had received chemotherapy for the treatment of primary tumors. Furthermore, all of them had taken chemotherapy for liver metastases and they had been accepted as refractory to chemotherapy. Partial hepatectomy, chemoembolization and radiofrequency ablation treatment had been performed in 2, 2 and 6 patients respectively. The treatment was repeated at least one more time in 5 patients to the same or other lobes.

All the patients underwent liver function tests and dynamic liner MRI as well as basal F18-FDG PET/CT examination before the treatment. The first control F18-FDG PET/CT scan was undergone by all patients 6 weeks after treatment for the evaluation of treatment response.

The patients were divided into two groups according to the disease stage; those with only liver metastases (H) and those with metastases in other organs (EH).

### Selective intraarterial radionuclide therapy (SIRT)

In all patients, widely accepted parameters regarding liver reserve, bone marrow reserve (granulocytes > 1500/μL, platelets > 60000/μL), and hepatic vascularity were used as inclusion and exclusion criteria. Liver reserve was evaluated using bilirubin, aspartate transaminase (AST), alanine transaminase (ALT), and alkaline phosphatase (ALP) levels in blood. A bilirubin level < 2 mg/dl and AST/ALT/ALP levels less than 5 times the normal upper limit were required for radioembolization. 10 patients did not receive the therapy according to these criteria. Patients with ascites, portal hypertension, portal venous thrombosis or an expected survival < 3 months were excluded as well as the patients with contraindications for angiography and selective visceral catheterization. To evaluate vascular tree, a therapy-planning angiogram was performed. With this angiogram, branches of hepatic artery to the gastrointestinal tract were coiled to prevent Y-90 reflux to the stomach, i.e. to gastro-duodenal artery and right gastric artery. At the end of this planning angiogram, a 150 MBq dose of ^99m^Tc-labelled macroaggregated albumin (MAA) was administered through the catheter in an attempt to detect arteriovenous shunts from the hepatic arterial system to the pulmonary system or gastrointestinal tract. After this procedure, gamma imaging was obtained and regions of interest were drawn around the liver and lungs in anterior planar images, and the pulmonary shunt was calculated using the following equation: pulmonary shunt fraction = ROI _lung counts_/(ROI _lung counts _+ ROI _liver counts_. Patients with a pulmonary shunt less than 20% were eligible for therapy. 2 patients were excluded because of the pulmonary shunt higher than 20%. In 78 patients who were suitable for therapy, the Y-90 dose was adjusted according to the following body surface area method: activity (GBq) = (BSA - 0.2) + tumor volume/total liver volume. The Y-90 resin microspheres (Sirtex Medical, Australia) were injected through the hepatic artery catheter under intermittent fluoroscopic visualization. Within 1 to 24 hours after microsphere infusion, Bremsstrahlung images were obtained to confirm that the Y-90 was deposited only in the liver. All patients were hospitalized overnight and medications like analgesics, antiemetic, and H_2 _antagonist were administered, if necessary. All patients were closely monitored until acute or late toxicities were resolved.

### PET/CT imaging

PET/CT images were acquired with GE Discovery ST PET/CT scanner. During imaging patients were required to have at least 6 hours fasting and checked if their blood glucose levels were under 150 mg/dl. Oral contrast agents were applied to all patients. Images were obtained while patients were lying in a supine position from vertex to proximal femur. Whole body F18-FDG PET/CT imaging was performed approximately 1 hour after an intravenous injection of 8-10 mCi FDG. During the waiting period patients rested in a quiet room without receiving muscle relaxant. PET images were acquired for 4 minutes per bed position. Emission PET images were reconstructed with non-contrast CT. A CT image was obtained from the patient's integrated F18-FDG PET/CT with the use of a standardized protocol involving 140 kV, 70 mA, a tube rotation time of 0.5 s per rotation, a pitch of 6 and a section thickness of 5 mm. Patients were allowed to breathe normally during procedure. Attenuation-correction was done by PET/CT fusion images on three planes (trans-axial, coronal and sagittal) and were reviewed on Xeleris Workstation (GE Medical System). F18-FDG PET/CT images were evaluated visually and semi-quantitatively by two experienced nuclear medicine specialists. The number, location and SUV values of liver lesions were recorded.

### Evaluation of treatment response

Metabolic treatment response evaluated by PET/CT in the 6^th ^week after treatment. FDG-PET/CT was repeated in per six weeks periods. The response criterion had been described as at least 20% decrease of SUV value. Also in patients with neuroendocrine tumor serial Ga-68 PET/CT was used for evaluation of response. Patients were divided into 2 groups according to their treatment response (R = responder, NR = non-responder).

### Statistical analysis

According to R, NR, H and EH groups, overall survival analysis was performed using Kaplan-Meier method and comparison was done using the log rank (Mantel-Cox) test. SPSS version 15.0 was used for statistical analysis. Statistical significance was as accepted p < 0.05.

## Results

### Patients

78 patients (49 M; 29 F; mean age: 62.4 ± 2.3 years) received intraarterial radionuclide therapy with Y-90 microspheres for liver metastasis or primary HCC between June 2006 and October 2010. Although 25 patients had primary HCC diagnosis, the remainder had unresectable multiple liver metastases of different cancers (35 colorectal, 7 gastric, 4 breast, 1 pancreas, 1 renal cell, 1 esophagus cancer, 3 neuroendocrine tumor and 1 malignant melanoma).

### Radiation Delivery

68 patients received treatment for the right lobe, seven patients received treatment for the left lobe and 3 patients for both lobes. The mean treatment dose was estimated at 1.62 GBq (range: 1-1.8 GBq). In all patients, the leakage to the lungs was less than 20%. Therefore, neither reduction in the estimated dose nor discontinuation of the treatment was required.

### Toxicity

The technical success of the intraarterial delivery of Y-90 microspheres was 100% and none of the patients experienced complications due to angiographic intervention. All patients experienced post-radioembolization syndrome characterized by mild abdominal pain, nausea, and sub-febrile fever. A combination of a non-opioid analgesic, an antiemetic and a H_2 _receptor blocker was given to patients not tolerating these symptoms. Symptoms decreased in intensity within one week and completely disappeared within 15 days. No difference has been found in complication rates between the two lobes. Bremsstrahlung imaging done 24 hours after treatment did not show any activity outside the liver. All patients were hospitalized for one night as a preventive measure and prolonged hospitalization was not required by any of the patients.

### Response

**Figure 1:**
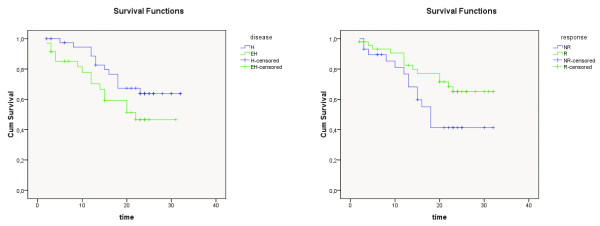
**Cumulative survival curves of the R, NR, H and EH subgroups in the whole patient group**. Time: months, R: responder, NR: nonresponder, H: hepatic, EH: extrahepatic.

**Figure 2:**
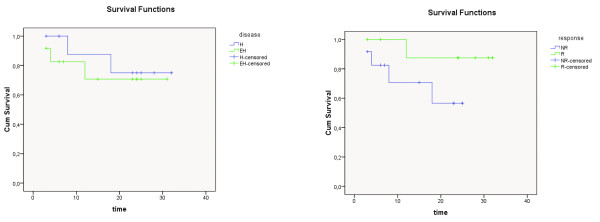
**Cumulative survival curves of the R, NR, H and EH subgroups in the colorectal group**. Time: months, R: responder, NR: nonresponder, H: hepatic, EH: extrahepatic.

**Figure 3:**
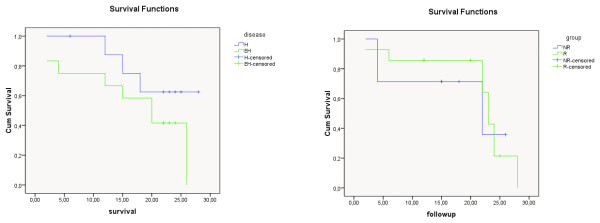
**Cumulative survival curves of the R, NR, H and EH subgroups in the HCC group**. Survival: months, R: responder, NR: nonresponder, H: hepatic, EH: extrahepatic.

In the evaluation of treatment response; 43 (55%) patients were responder (R) (Figure 1, 2, 3) and 35 (45%) patients were non-responder (NR) in the sixth week F18-FDG PET/CT. Mean pretreatment SUVmax value of R group was 11.6 and NR group was 10.7. While only 11 (31%) out of 35 NR patients had H disease, 30 (69%) out of 43 R patients had H disease (p < 0.05) (Table 1).

**Table 1:** H and EH disease rates of the R and NR groups.

No of patients (%)	R	NR
H	30(69%)	11(31%)

EH	13(31%)	24(69%)

### Survival analysis

**Figure 4:**
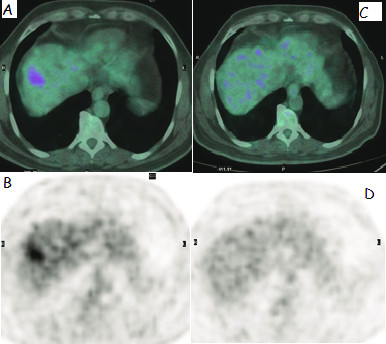
**60 years-old male patient who took 1.2GBq Y-90 microsphere therapy to the right lobe of the liver for HCC**. 4A, 4B: axial- fused and PET images of the liver before the treatment. 4C, 4D: axial- fused and PET images of the liver after the treatment.

**Figure 5:**
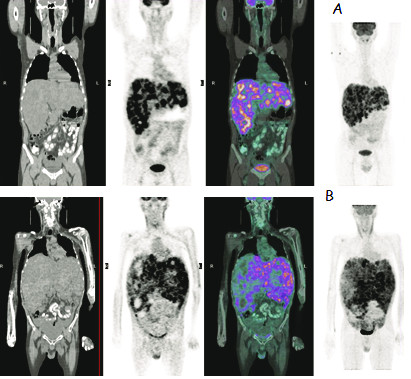
**39 years-old male patient who received 1.6 GBq Y-90 microsphere therapy to the right and left lobe in separate sessions for primary hemangioendothelioma of the liver**. 5A; coronal CT, 18F-FDG PET, fused and maximum intensity projection images of the whole body before the treatment. 5B; coronal CT, 18F-FDG PET, fused and maximum intensity projection images of the whole body after the treatment.

**Figure 6:**
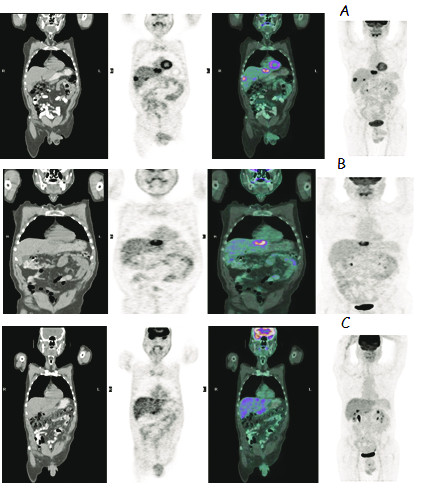
**54 years-old male patient who received 1.7 GBq Y-90 microsphere therapy to the right and left lobes in separate sessions for liver metastases of colorectal cancer**. 6A; coronal CT, 18F-FDG PET, fused and maximum intensity projection images of the whole body before the treatment. 6B; coronal CT, 18F-FDG PET, fused and maximum intensity projection images of the whole body after the treatment of the right lobe. 6C; coronal CT, 18F-FDG PET, fused and maximum intensity projection images of the whole body after the treatment of the left lobe.

The mean overall survival time of R group was calculated as 25.63 ± 1.52 months and NR group's 20.45 ± 2.11 (p = 0.04) (Table 2, 3). The mean overall survival time of H group was computed as 25.66 ± 1.52 months and EH group's 20.76 ± 1.97 (p = 0.09) (Table 4, 5). The survival curves of the whole patient group, the colorectal group and the HCC group, according to the treatment response and disease stage were demonstrated in Figure 4, 5 and 6, respectively.

**Table 2:** The mean and median survival times of the R and NR groups.

		Means and Medians for Survival Time						
		**Mean^a^**				**Median**		

			**95% Confidence Interval**				**95% Confidence Interval**	

**Response**	**Estimate**	**Std. Error**	**Lower Bound**	**Upper Bound**	**Estimate**	**Std. Error**	**Lower Bound**	**Upper Bound**

NR	20,452	2,116	16,305	24,598	18,000	1,495	15,070	20,930

R	25,637	1,523	22,652	28,622	-	-	-	-

Overall	23,654	1,279	21,147	26,161	-	-	-	-

^a. ^Estimation is limited to the largest survival time if it is censored.

**Table 3:** Mantel-cox overall comparison of the R and NR groups.

Overall Comparisons			
	**Chi-Square**	**df**	**Sig**.

Log Rank (Mantel-Cox)	3,915	1	,048

Test of equality of survival distributions for the different levels of response.

**Table 4:** The mean and median survival times of the H and EH groups.

		Means and Medians for Survival Time						
		**Mean^a^**				**Median**		

			**95% Confidence Interval**				**95% Confidence Interval**	

**Disease**	**Estimate**	**Std. Error**	**Lower Bound**	**Upper Bound**	**Estimate**	**Std. Error**	**Lower Bound**	**Upper Bound**

H	25,666	1,525	22,678	28,655	-	-	-	-

EH	20,769	1,971	19,906	24,633	22,000	-	-	-

Overall	23,654	1,279	21,147	26,161	-	-	-	-

^a. ^Estimation is limited to the largest survival time if it is censored.

**Table 5:** Mantel-cox overall comparison of the H and EH groups.

Overall Comparisons			
	**Chi-Square**	**df**	**Sig**.

Log Rank (Mantel-Cox)	2,768	1	,096

Test of equality of survival distributions for the different levels of disease.

## Discussion

As mainly HCC, colorectal cancer and neuroendocrine tumors; SIRT has been used for the treatment of liver metastasis of several tumors and primary hepatocellular cancer. There have been different results in literature about the success of SIRT in liver metastasis of different tumors. It has been reported that the efficiency of SIRT in liver metastases of colorectal cancer was 90% in first-line therapy and 80% in second-line therapy [9]. In our patient group, we detected a rate of response as 55%. This rate might appear low, but from a recent study, we accepted a different response criterion as a 20% decrease in SUV levels of liver lesions. Our patient group also included 78 patients with different malignancies. The biological behavior of liver metastases of different tumor might vary. Furthermore all the patients received SIRT as a salvage therapy. Our response rate might have been affected for these reasons.

We preferred F18-FDG PET/CT for staging before the treatment and evaluation of treatment response. There are many advantages of F18-FDG PET/CT in the early stage after therapy. Firstly, it is known that F18-FDG PET/CT is more successful than conventional imaging methods in evaluating treatment response at the early period after SIRT [8,10]. Also Wong et al. have reported that there is a correlation between liver tumor burden and the presence of extra-hepatic disease detected by PET/CT before Y-90 microspheres treatment [11]. So, F18-FDG PET/CT may provide extra information in predicting the development of extra-hepatic disease.

In different studies, the survival times after Y-90 microsphere treatment of liver metastases had been reported between 6.7 and 17.0 months [12-20]. These periods may change according to the microsphere type used, previous chemotherapy regimens and patient groups. For this reason, it would be an optimal approach to make a comparison with an age, diagnosis, stage and chemotherapy matched control group. Since in our study, the treatment was applied as a salvage protocol to most of the patients, it is very difficult to find a control group which has patients with same diagnosis and same stage of disease. For this reason we compared the survival times of our groups to current literature. It has been calculated that mean survival times of R and NR groups as 25.63 ± 1.52 and 20.45 ± 2.11 months (p = 0.04) respectively. Because the difference between the two groups was statistically significant, SIRT is seem to be beneficial in the treatment of liver tumors. However this study is a retrospectively designed study which has small heterogeneous patient number, new prospective randomized studies are needed to support this result. Also results of this study support the conclusion which is that FDG PET/CT is a useful method for evaluating treatment response in patients who have undergone SIRT for liver metastasis. In the subgroup analysis; mean overall survival time of colorectal patients group was found to be 20.5 months while the R and NR groups' were 21.35 and 18.28 months respectively. In the HCC group; the mean overall survival, R and NR groups' survival times were 25.8, 18.24 and 29.5 months respectively.

The treatment response was also evaluated according to the disease stage with H and EH groups. The mean overall survival time of the H group was computed as 25.66 ± 1.52 months and EH group's 20.76 ± 1.97 (p = 0.09). The difference between the two groups was not statistically significant but it was very close to the limit of p = 0.05. In the subgroup analysis of colorectal patients group, the mean survival time of H and EH groups were 23.12 and 17.08 months respectively. In the HCC group; the H and EH groups' survivals were 27.2 and 23.9 months respectively. In the separate evaluation of patients according to diagnosis, the difference between the R and NR groups and E and EH groups was not statistically significant. This result could be related to the fact that the numbers of each patient group were small in the separated analysis. For this reason, larger prospective randomized new studies are needed.

## Conclusion

SIRT is a useful treatment method which can contribute to the lengthening of survival times in patients with primary or metastatic unresectable liver malignancies. Also F18-FDG PET/CT is seen to be a successful imaging method in evaluating treatment response for predicting survival times in this patient group.

## Competing interests

The authors declare that they have no competing interests.

## Authors' contributions

CS and EO data collection. NOK drafted the manuscript. SL, CS and SB participated in the design of the study and performed the statistical analysis. CS, NOK conceived of the study, and participated in its design and coordination. All authors read and approved the final manuscript.

## References

[B1] Gray B Van Hazel G Hope M Burton M Moroz P Anderson J Gebski V Randomised trial of SIR-Spheres plus chemotherapy vs. chemotherapy alone for treating patients with liver metastases from primary large bowel cancer Ann Oncol 2001 12 12 1711 20 10.1023/A:1013569329846 11843249

[B2] Stubbs RS Wickremesekera SK Selective internal radiation therapy (SIRT): a new modality for treating patients with colorectal liver metastases HPB (Oxford) 2004 6 3 133 9 10.1080/13651820410025084 PMC2020675 18333066

[B3] Herba MJ Thirlwell MP Radioembolization for hepatic metastases Semin Oncol 2002 29 2 152 9 10.1053/sonc.2002.31672 11951213

[B4] Houle S Yip TK Shepherd FA Rotstein LE Sniderman KW Theis E Cawthorn RH Richmond-Cox K Hepatocellular carcinoma: pilot trial of treatment with Y-90 microspheres Radiology 1989 172 3 857 60 2549567 10.1148/radiology.172.3.2549567

[B5] Lau WY Ho S Leung TW Chan M Ho R Johnson PJ Li AK Selective internal radiation therapy for nonresectable hepatocellular carcinoma with intraarterial infusion of 90yttrium microspheres Int J Radiat Oncol Biol Phys 1998 40 3 583 92 10.1016/S0360-3016(97)00818-3 9486608

[B6] Blanchard RJ Morrow IM Sutherland JB Treatment of liver tumors with yttrium-90 microspheres alone Can Assoc Radiol J 1989 40 4 206 10 2766018

[B7] Yan ZP Lin G Zhao HY Dong YH An experimental study and clinical pilot trials on yttrium-90 glass microspheres through the hepatic artery for treatment of primary liver cancer Cancer 1993 72 11 3210 5 10.1002/1097-0142(19931201)72:11<3210::AID-CNCR2820721113>3.0.CO;2-6 8242543

[B8] Bienert M McCook B Carr BI Geller DA Sheetz M Tutor C Amesur N Avril N 90Y microsphere treatment of unresectable liver metastases: changes in 18F-FDG uptake and tumour size on PET/CT Eur J Nucl Med Mol Imaging 2005 32 7 778 87 Epub 2005 Mar 17 10.1007/s00259-004-1752-1 15772860

[B9] Vente MA Wondergem M van der Tweel I van den Bosch MA Zonnenberg BA Lam MG van Het Schip AD Nijsen JF Yttrium-90 microsphere radioembolization for the treatment of liver malignancies: a structured meta-analysis Eur Radiol 2009 19 4 951 9 Epub 2008 Nov 7 10.1007/s00330-008-1211-7 18989675

[B10] Szyszko T Al-Nahhas A Canelo R Habib N Jiao L Wasan H Pagou M Tait P Assesment of response to treatment of unresectable liver tumours with Y-90 microspheres: value of FDG PET versus computed tomography Nucl Med Comm 2007 28 15 20 10.1097/MNM.0b013e328011453b 17159544

[B11] Wong CY Gates VL Tang B Campbell J Qing F Lewandowski RJ Thie J Ho CL Savin M Salem R Fluoro-2-Deoxy-D-Glucose positron emission tomography/Computed tomography predicts extrahepatic metastatic potential of colorectal metastasis: a practical guide for yttrium-90 microsphere liver-directed theraphy Cancer Bioth and Radioph 2010 25 233 236 10.1089/cbr.2009.0735 20423237

[B12] Stubbs RS Cannan RJ Mitchell AW Selective internal radiation therapy (SIRT) with 90Yttrium microspheres for extensive colorectal liver metastases Hepatogastroenterology 2001 48 38 333 7 11379303

[B13] Van Hazel G Blackwell A Anderson J Price D Moroz P Bower G Cardaci G Gray B Randomised phase 2 trial of SIR-Spheres plus fluorouracil/leucovorin chemotherapy versus fluorouracil/leucovorin chemotherapy alone in advanced colorectal cancer J Surg Oncol 2004 88 2 78 85 10.1002/jso.20141 15499601

[B14] Murthy R Xiong H Nunez R Cohen AC Barron B Szklaruk J Madoff DC Gupta S Wallace MJ Ahrar K Hicks ME Yttrium 90 resin mircospeheres for the treatment of unresectable colorectal hepatic metastases after failure of multiple chemotherapy regimens: preliminary results J Vasc Interv Radiol 2005 16 937 945 16002501 10.1097/01.RVI.0000161142.12822.66

[B15] Stubbs R O'Brien I Correia M Selective internal radiation therapy with 90Y spheres with colorectal liver metastases: singe-cetre experience with 100 patients ANZJ Surg 2006 79 696 703 10.1111/j.1445-2197.2006.03834.x 16916386

[B16] Anderson JH Goldberg JA Bessent RG Kerr DJ McKillop JH Stewart I Cooke TG McArdle CS Glass yttrium-90 microspheres for patients with colorectal liver metastases Radiother Oncol 1992 25 137 139 10.1016/0167-8140(92)90020-U 1438931

[B17] Andrews JC Walker SC Ackermann RJ Cotton LA Ensminger WD Shapiro B Hepatic radioembolization with yttrium-90 containing glass micro-spheres: preliminary results and clinical follow-up J Nucl Med 1994 35 1637 1644 7931662

[B18] Sato KT Lewandowski RJ Mulcahy MF Atassi B Ryu RK Gates VL Nemcek AA Jr Barakat O Benson A Mandal R Talamonti M Wong CY Miller FH Newman SB Shaw JM Thurston KG Omary RA Salem R Unresectable chemorefractory liver metastases: radioembolization with Y-90 microspheres-safety, efficacy and survival Radiol 2008 248 507 515 10.1148/radiol.2472062029 18349311

[B19] Bujold A Dawson LA Stereotactic radiation therapy and selective internal radiation therapy for hepatocellular carcinoma Cancer Radiother 2011 15 1 54 63 Epub 2011 Jan 15 21239204 10.1016/j.canrad.2010.11.003

[B20] Kennedy AS Salem R Radioembolization (yttrium-90 microspheres) for primary and metastatic hepatic malignancies Cancer J 2010 16 2 163 75 Review 10.1097/PPO.0b013e3181d7e8cf 20404614

