# Selective intraarterial radionuclide therapy with Yttrium-90 (Y-90) microspheres for unresectable primary and metastatic liver tumors

**DOI:** 10.1186/1477-7819-9-120

**Published:** 2011-10-06

**Authors:** N Ozlem Kucuk, Cigdem Soydal, Seda Lacin, Elgin Ozkan, Sadık Bilgic

**Affiliations:** 1 Ankara University, Medical School, Department of Nuclear Medicine, Ankara, Turkey; 2 Ankara University, Medical School, Department of Radiology, Ankara, Turkey

## Correction

After the publication of this work [1], we became aware of the fact that there was date mistakes in the Abstract, Patients and method and Results sections. The correct text is included below.

In the '**Methods**' section of the Abstract, the first sentence should have read:

"The clinical and follow-up data of 124 patients who were referred to our department for SIRT between June 2008 and October 2010 were evaluated retrospectively."

In The '**Patients**' section of the Patients and Method, the first sentence should have read:

"The clinical and follow-up data of 124 patients who were referred to our department for SIRT between June 2008 and October 2010 were evaluated retrospectively."

In the '**Patients**' section of the Results, the first sentence should have read:

"78 patients (49 M; 29 F; mean age: 62.4+2.3 years) received intra-arterial radionuclide treatments with 90 Y for liver metastasis or primary HCC between June 2008 and October 2010."
